# Comparison of genetic variation between rare and common congeners of *Dipodomys* with estimates of contemporary and historical effective population size

**DOI:** 10.1371/journal.pone.0274554

**Published:** 2022-09-13

**Authors:** Michaela K. Halsey, John D. Stuhler, Natalia J. Bayona-Vásquez, Roy N. Platt, Jim R. Goetze, Robert E. Martin, Kenneth G. Matocha, Robert D. Bradley, Richard D. Stevens, David A. Ray

**Affiliations:** 1 Department of Biological Sciences, Texas Tech University, Lubbock, Texas, United States of America; 2 Department of Natural Resources Management, Texas Tech University, Lubbock, Texas, United States of America; 3 Department of Environmental Health Science, University of Georgia, Athens, Georgia, United States of America; 4 Institute of Bioinformatics, University of Georgia, Athens, Georgia, United States of America; 5 Texas Biomedical Research Institute, San Antonio, Texas, United States of America; 6 Natural Sciences Department, Laredo College, Laredo, Texas, United States of America; 7 Department of Biology, McMurry University, Abilene, Texas, United States of America; 8 Department of Biology, South Arkansas Community College, El Dorado, Arkansas, United States of America; 9 Natural Science Research Laboratory, Museum of Texas Tech, Lubbock, Texas, United States of America; Manaaki Whenua: Landcare Research New Zealand, NEW ZEALAND

## Abstract

Species with low effective population sizes are at greater risk of extinction because of reduced genetic diversity. Such species are more vulnerable to chance events that decrease population sizes (e.g. demographic stochasticity). *Dipodomys elator*, (Texas kangaroo rat) is a kangaroo rat that is classified as threatened in Texas and field surveys from the past 50 years indicate that the distribution of this species has decreased. This suggests geographic range reductions that could have caused population fluctuations, potentially impacting effective population size. Conversely, the more common and widespread *D*. *ordii* (Ord’s kangaroo rat) is thought to exhibit relative geographic and demographic stability. We assessed the genetic variation of *D*. *elator* and *D*. *ordii* samples using 3RAD, a modified restriction site associated sequencing approach. We hypothesized that *D*. *elator* would show lower levels of nucleotide diversity, observed heterozygosity, and effective population size when compared to *D*. *ordii*. We were also interested in identifying population structure within contemporary samples of *D*. *elator* and detecting genetic variation between temporal samples to understand demographic dynamics. We analyzed up to 61,000 single nucleotide polymorphisms. We found that genetic variability and effective population size in contemporary *D*. *elator* populations is lower than that of *D*. *ordii*. There is slight, if any, population structure within contemporary *D*. *elator* samples, and we found low genetic differentiation between spatial or temporal historical samples. This indicates little change in nuclear genetic diversity over 30 years. Results suggest that genetic diversity of *D*. *elator* has remained stable despite reduced population size and/or abundance, which may indicate a metapopulation-like system, whose fluctuations might counteract species extinction.

## Introduction

Measuring genetic variation in rare, threatened, endemic, or endangered species has important implications for management and is integral to conservation efforts [1]. Population genetic summary statistics can be used to delimit management units based on significantly different allele frequencies [2], identify population structure [3], or assess connectivity of demographically disparate subpopulations [4]. One such critical measure for small populations is effective population size, N_e_ [5], the number of individuals in a population that would resemble an idealized population that would result in the same rate of inbreeding as the study population. This value can be influenced by fluctuations in census size, mating strategy, biased sex ratios, migration, demographic history, spatial dispersion, and population structure [6–9], and typically is far less than the census size. However, the N_e_ value is often hard to interpret without context. One such context to help understand the potential impacts of fluctuations of N_e_ is comparison between more restricted, possibly threatened species and a widespread congener, which are presumed to harbor more genetic variation.

The Texas kangaroo rat (*Dipodomys elator*) is a monotypic, heteromyid rodent that has a limited distribution in north-central Texas [10–14]. Though previously found in two counties in Oklahoma, it appears to have been extirpated from that state [15]. Moreover, *D*. *elator* has a small geographic range and a presumably low dispersal capability [16, 17], which increases isolation from nearby subpopulations. *D*. *elator* is listed as “vulnerable” by the International Union for Conservation of Nature and Natural Resources [18].

The distribution of the Texas kangaroo rat appears dynamic [19]. For instance, though the species was described from a specimen in Clay County in 1894 [20], it has not been observed there in over 60 years. Resampling of sites where it has been previously documented have failed to detect the species, and new localities of presence have been identified in contemporary surveys [21]. Previous studies of *D*. *elator* population genetics have detected population structure and limited diversity [22, 23] and are relevant in assessing genetic diversity within the species. Hamilton et al. 1987 reported low values of genetic distance between *D*. *elator* populations in Wichita, Wilbarger, and Hardeman counties and suggested gene flow or short time of separation between populations keeping those values low. Pfau et al. 2019 [23] concluded with the study of mitochondrial DNA and microsatellites, that genetic drift, and not gene flow has had a greater impact on to the genetic structure on the species in Texas, but that the impact of genetic drift was minimal over 17–36 years evaluated. These two studies relied on few molecular markers (i.e. enzymes, mtDNA genes, and microsatellites) but established valuable reference points for our study.

Ord’s kangaroo rat, *D*. *ordii* is a medium sized rodent that occurs from Canada into Mexico. Of the 34 subspecies, only one, *D*. *ordii richardsoni*, is present in the same region as *D*. *elator* [24]. Given its large geographic range and preferred commonly available habitat choice (i.e. sandy soils), *D*. *ordii* is not listed on any state or U.S. federal critically threatened and endangered lists. The population in Canada, however, is listed as endangered [25]. To our knowledge, there has not been a range-wide genetic analysis of *D*. *ordii*, and the last regional genetic study on *D*. *ordii* isoenzymes was published by Beck et al. 1981 [26]. It was found by Beck et al. 1981 [26] that *D*. *ordii* in the South Canadian River floodplain in Oklahoma were highly similar in terms of protein variation, with eight of 14 alleles examined being monomorphic.

Sequencing DNA from contemporary samples and from historical museum collections enables comparison of genetic diversity and population structure at different time periods [27, 28]. From such surveys, researchers can gain insight into historical demographics, which can be useful in understanding the underlying factors of genetic diversity. Fortunately, *D*. *elator* population surveys are complemented with genetic assays, either from collected specimens [12, 14] or prior genetic analysis [22], setting up critical comparisons of the species across space and time.

Here, we compare population genetic parameters of *D*. *elator* with *D*. *ordii* and compare *D*. *elator* samples from two time periods (1986–1995 and 2015–2017), and for recent samples, investigate differences in genetic diversity across the distribution. We make several predictions: 1) *D*. *elator* will exhibit a lower effective population size than *D*. *ordii*, and concomitantly, lower nucleotide diversity, lower observed heterozygosity, and higher inbreeding coefficients; 2) there will be greater genetic diversity among contemporary samples of *D*. *elator* than in historical samples, as contemporary samples were taken from across the distribution, compared to historical samples collected from three counties in the middle of its distribution; and 3) historical N_e_ from a coalescent approach for *D*. *ordii* and *D*. *elator* will demonstrate that *D*. *elator* will show a historical decline in N_e_, while *D*. *ordii* will show stable or increasing N_e_.

## Methods

### Sample collection

**Fig 1:**
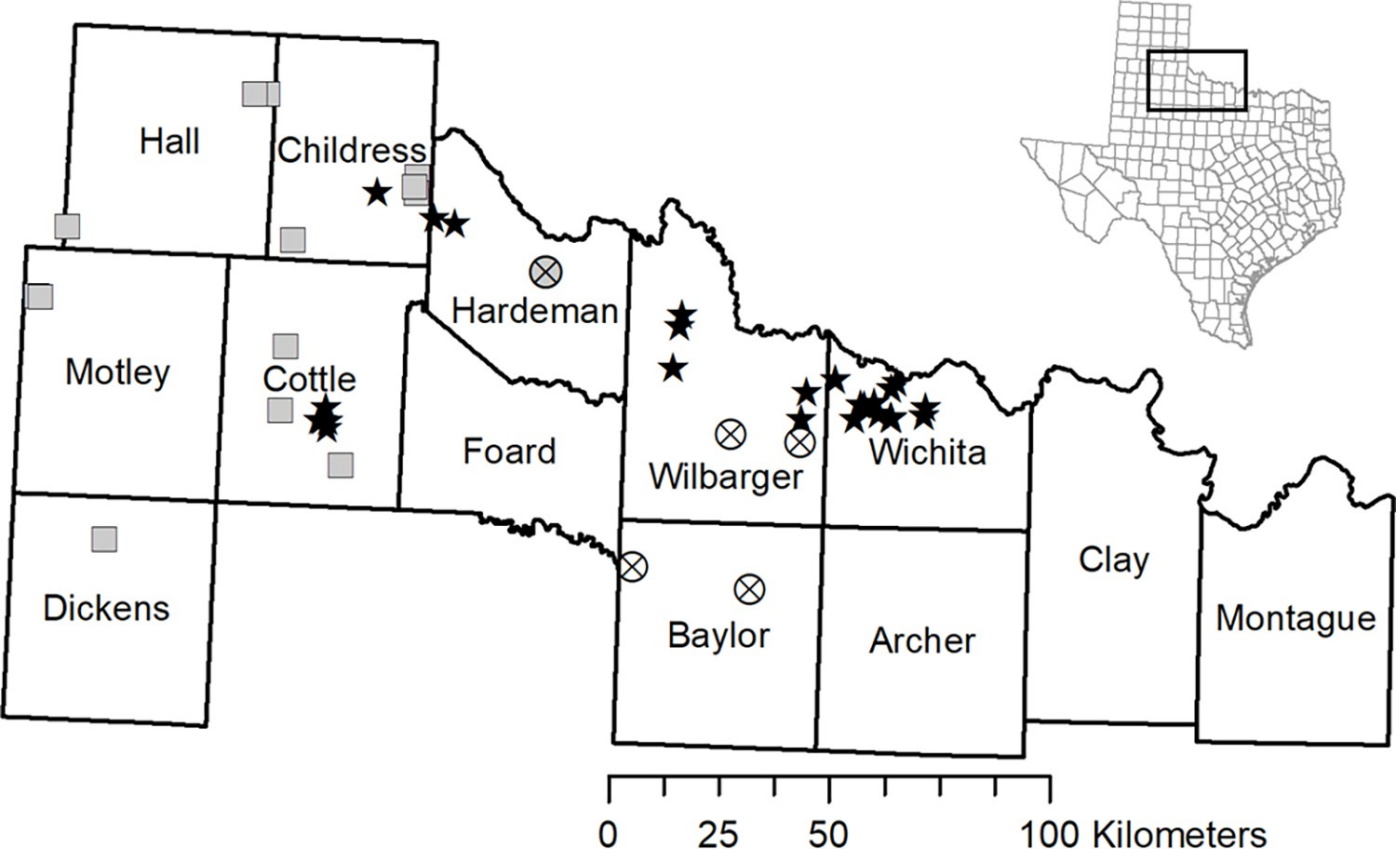
Map of kangaroo rat samples used in this study. Filled stars indicate contemporary *Dipodomys elator* samples whereas circles with an ‘x’ are historical *D*. *elator* samples. Twenty-eight historical samples in Hardeman are represented by one filled circle with an ‘x’. Filled squares represent *D*. *ordii* samples used in the study. Note the contemporary sampling gap located in Foard County, most of Hardman County, and in south Wilbarger County. Trapping restrictions and topography prevented collections in those regions. *D*. *elator* is thought to be extirpated in all counties except Cottle, Childress, Hardeman, Wilbarger, and Wichita counties. Map was constructed using ArcMap v10.8.1 and the 1:1,000,000-Scale National Boundaries of the United States data from the USGS EROS (Earth Resources Observatory and Science) Center).

Animal handling protocols were approved by the Institutional Animal Care and Use Committee at Texas Tech University (#T14083) and the Texas Parks and Wildlife Department (TPWD #SPR-1013-154). Kangaroo rats were captured using Sherman live traps (23x9x8 cm; H.B. Sherman Traps, Inc. Tallahassee, Florida) during surveys within the historical range of *D*. *elator* (Fig 1) from 2015–2017. When a *D*. *elator* individual was captured, it was either 1) taken as a voucher specimen for deposition at the Natural Science Research Laboratory (NSRL) at the Museum of Texas Tech University, in which DNA from the liver was used or, 2) had between two to four whiskers extracted from either side of the rostrum [29]. In the latter case, thicker whiskers (i.e., macrovibrissae) were selected with the follicle intact. Whiskers were stored in a sterile vial with 1% sodium dodecyl sulfate (SDS) lysis buffer [30]. A buccal swab was also collected from one *D*. *elator* individual as described in detail in Halsey et al. 2021 [29].

Other methods of collecting DNA from rats included tail salvages and from toe clips from museum specimens (S1 Table). *D*. *elator* tail lengths average about 196 mm [31] and at times the end of the tail (i.e., the plume) was severed by the door of an activated Sherman trap. These salvaged tail plumes were placed in sterile vials of 1% sodium dodecyl sulfate (SDS) lysis buffer [31]. Toe clips samples had been collected *in situ* from rats from 1986–1995 as part of a population survey of the species by REM and KGM. These samples were not suspended in any buffer and were kept at around -20°C to prevent degradation of DNA.

In total, 70 *D*. *elator* samples were analyzed from one of five tissue types (i.e, liver, whisker, tail, buccal swab, and toe clips) and two time periods (historical collections from 1986–1995 and contemporary surveys from 2015–2017; S1 Table and S1 File). *D*. *elator* liver samples were collected within the contemporary time frame, and animals were euthanized using isoflurane. Additionally, 26 *D*. *ordii* liver samples were collected in five counties from 2015–2017. Contemporary sampling followed guidelines established by the American Society of Mammalogists [32].

Throughout the manuscript the *D*. *elator* samples will be referred to using the following descriptors: ‘historical’, from 19861995; ‘contemporary’, from 20152017; ‘west’, collected from Cottle, Childress, or Hardeman counties; and ‘east’ collected from Baylor, Wilbarger, and Wichita counties (Fig 1). Individuals noted as from the *“*sampling gap” are historical specimens that were captured in an area that is inaccessible or not detected in contemporary surveys.

### DNA extraction

DNA was extracted using the Qiagen DNeasy Blood and Tissue spin column protocol (Qiagen; Venlo, Netherlands). For liver, toe clips, and tail salvages, the manufacturer’s recommendations were followed. Using a protocol similar to Moraes-Barros et al. 2007 [33], instruments were autoclaved, and work area was sterilized using 50% bleach and 70% ethanol prior to DNA isolation to prevent contamination. Toes were washed with MilliQ® water. With sterilized scissors, the claw was separated from the rest of the toe and properly discarded. The declawed toe was then placed in a 1.5 mL centrifuge tube with 180 μL buffer TE and 20 μL proteinase-K and incubated overnight. For whisker and buccal swab samples, the protocol found in Halsey et al. 2021 [29] was implemented. In all cases, DNA concentration was fluorometrically quantified using the Qubit 3.0, high sensitivity assay (Invitrogen, Life Technologies, Carlsbad, CA).

### 3RAD library prep, sequencing, and data processing

RADseq libraries were prepared following the 3RAD protocol found in Bayona-Vásquez et al. 2019 [34]. Details of library prep conditions used in this study are provided in S2 File. In short, restriction enzyme combinations were tested in a subset of samples from both species, and according to digestion patterns and pilot sequence data, the best combination (i.e. MspI, EcoRI, and ClaI), was further used for all samples. DNA were normalized, digested, enzyme-specific adapters were ligated, and ligation products were purified. To generate full- length library constructs, ligated products were amplified using iTru5 and iTru7 primers [35]. A molecular ID tag (iTru 5 8N) was incorporated in the first cycle of PCR for detection of PCR duplicates [34, 36]. PCR products were purified, pooled, and size-selected at a range of 550 bp +/- 15%. Size-selected fragments were purified and sequenced using an Illumina HiSeq 3000 to generate 150 bp paired end data at Oklahoma Medical Research Foundation Genomics Core or Novogene Inc.

Stacks v1.48 and v2.01 [37] was employed to demultiplex, analyze, and export data into other formats. After demultiplexing, poor reads were filtered using the AfterQC ‘after.py’ pipeline [38]. Poor reads were defined as exhibiting a low-quality score (PHRED score < 15), bad overlaps (i.e., mismatched reads), too many ambiguous nucleotides (greater than 40% of the read), short read lengths (< 35 base pairs), or homopolymer regions. If a read failed one of these criteria, it was removed from downstream analyses. Reads (2 x 150 bp) were aligned within Stacks to the *D*. *ordii* genome assembly (accession ID GCA_000151885.1; BioProject ID PRJNA20385) using the Burrows-Wheeler aligner [39]. Aligned data are available at The National Center for Biotechnology Information (NCBI) Sequence Read Archive (SRA) under BioProject ID PRJNA766428.

Data were grouped into putative loci, and polymorphisms were identified with the ‘gstacks’ module in Stacks. Common population genetic statistics such as observed and expected heterozygosity, nucleotide diversity, and inbreeding coefficients were calculated using the ‘populations’ module. This step was conducted with four different missingness values (-r) to identify the most appropriate parameter setting. The -r value [37] has been shown to bias population genetic measures, especially in cases where data are not plentiful, and can influence biological implications [40–44]. We used ‘populations’ module using the 75% rule (-r 0.75), two liberal filters (-r of 0.25 and 0.5) and a more conservative filter (-r 0.95). For most downstream analyses, -r 0.75 was used as the main dataset. For other parameters, program defaults were used, including a maximum observed heterozygosity of 1 and a minor allele frequency of 0. These parameters were used for both contemporary and historical samples to reduce bias in summary statistic estimates due to different protocols. Also, as a final filtering step, loci and individuals that had greater than 20% missing data were removed using the ‘missingno’ function in the ‘poppr’ R package [45] for only the -r 0.75 dataset. Code for populations and additional filtering can be found in S3 File.

### Population genetics

For genetic diversity, observed and expected heterozygosity were calculated using the ‘summary’ function in the R package adegenet version 2.1.1 [46]. To assess population inbreeding and differentiation, F_IS_ and F_ST_ values were calculated using hierfstat [47] and an analysis of molecular variance or AMOVA [48]. Nei’s genetic distances [49] were determined and plotted using the ‘aboot’ function in the poppr R package [45]. Relatedness was calculated using the.relatedness function in vcftools [50].

### Estimation of effective population size

To determine effective population size using NeEstimator v2.1 [51], the Genepop file [52] generated by Stacks was used on our contemporary dataset. NeEstimator calculates N_e_ using three methods: linkage disequilibrium, molecular co-ancestry, and a temporal method. The first two methods were used to determine contemporary effective population sizes per species.

For historical N_e_ of *D*. *elator*, the Extended Bayesian Skyline Plot (EBSP) coalescent test as implemented in BEAST 2.0 [53] was used. Loci containing more than 3 single nucleotide polymorphisms were determined and only individuals with data for these loci were retained. The protocol outlined in Trucchii et al. 2014 [54] was followed, using a strict molecular clock set to 1.0 and a generation time of 3 years [55]. This same process was followed for contemporary *D*. *ordii* samples. However, only one individual from Dickens County (Fig 1) was included to avoid misinterpretations due to possible inbreeding since many individuals collected from that county were from a single location.

### Population structure

To infer population structure for *D*. *elator*, the STRUCTURE algorithm was used [56]. All singletons and private doubletons were removed, which have been shown to mask weak population structure [57, 58]. Only one randomly selected SNP from each locus was used to minimize possible effects of linked data. Allele frequencies were assumed to be correlated, and location prior settings were not used as they did not improve estimates. For all runs, 50,000 burn-in iterations were executed and 200,000 Markov Chain Monte Carlo (MCMC) repetitions with 3 replicates at each K, which ranged from 1–5. We used the Evanno method of delta K to determine the optimal value of “k” [59]. The program DISTRUCT v1.1 was used to visualize the final output of STRUCTURE analyses [60].

### Principal components analysis

To visualize genetic structure of the *D*. *elator* population without assigning individuals to clusters *a priori*, a Principal Components Analysis was conducted using the function dudi.pca in the R package ‘adegenet’ version 2.1.1 [46] on historical and contemporary samples. The first two axes explained almost 98% of the data and therefore were the only two axes retained. PCAs for samples separated by time periods are included in S4 File.

## Results

In all, 96 kangaroo rats were sequenced and analyzed from two species in eight counties in north-central Texas. Because these data were aligned using the *D*. *ordii* genome, it is expected that *D*. *elator* will show reduced differentiation estimates and fewer SNPs generated at a lower depth. 3RAD analysis for 70 individual *D*. *elator* samples produced over 34 million reads (mean = 5.24x10^5^, SD ± 4.60x10^5^). For *D*. *elator*, there were 33 samples for the historical dataset and 37 samples for contemporary dataset. Before filtering within the ‘populations’ module, there were 330,326 SNPs suitable for analysis. Approximately 1.5% of reads per sample were removed from the dataset following AfterQC filtering. After all filtering steps, 3,935 SNPs remained from 55 *D*. *elator* individuals (30 contemporary and 25 historical samples).

Similar analysis for the 26 *D*. *ordii* samples produced over 10 million reads (mean = 3.62x10^5^, SD ± 4.5x10^5^). Prior to ‘populations’ filtering, approximately 382,514 SNPs were eligible for further analysis. Fewer than 2% of reads per sample were removed from the dataset. Read depth and average number of reads per individual can be found in S2 Table. After all filtering steps, 11,963 SNPs remained from 13 individuals.

### Summary population genetics

Across the four -r values evaluated (0.25, 0.5, 0.75, 0.95), there were as few as 7 single nucleotide polymorphisms (SNPs) to as many as 61,000 SNPs to analyze (S3 Table). The general trend was that there were fewer SNPs analyzed as -r value increased and extreme values of -r (i.e. 0.25 and 0.95; S3 Table) yielded stronger deviations between observed and expected heterozygosity across all analyses.

When compared to each other using the -r 0.75 dataset contemporary *D*. *elator* samples showed lower levels of observed heterozygosity (0.041, SE±0.0002) than *D*. *ordii* (0.158, SE±0.125) which suggests lower genetic diversity in *D*. *elator*. Inbreeding coefficient F_IS_ was positive in all *D*. *elator* groups (0.015–0.031) except for in historical samples (-0.007). With an AMOVA, we found that F_ST_ values demonstrated low but significant genetic differentiation (0.044, p < 0.0001) between pairwise east and west samples. Based on climate, vegetation, edaphic, and land use characteristics across the study area [21], our a priori assumption was that there are two subpopulations (east and west).

### Current and historical effective population size

Only the linkage disequilibrium method in NeEstimator v.2.1 produced a value other than ‘Infinite’ for effective population size for *D*. *elator*. The estimated N_e_ of the east group was 171.3, with a 95% confidence interval (CI) of 158–186.9 using the lowest allele frequency (>0). For the west group, both the linkage disequilibrium and molecular co-ancestry methods returned ‘Infinite’ for N_e_ at all allele frequencies. In the historical Hardeman County dataset, in which all samples were from a single location, we estimate a N_e_ of 54.8 (CI = 53.7–56.0) using the lowest minor allele frequency (>0). No method with NeEstimator was able to provide an estimate of population size for *D*. *ordii*, other than ‘Infinite.’

**Fig 2:**
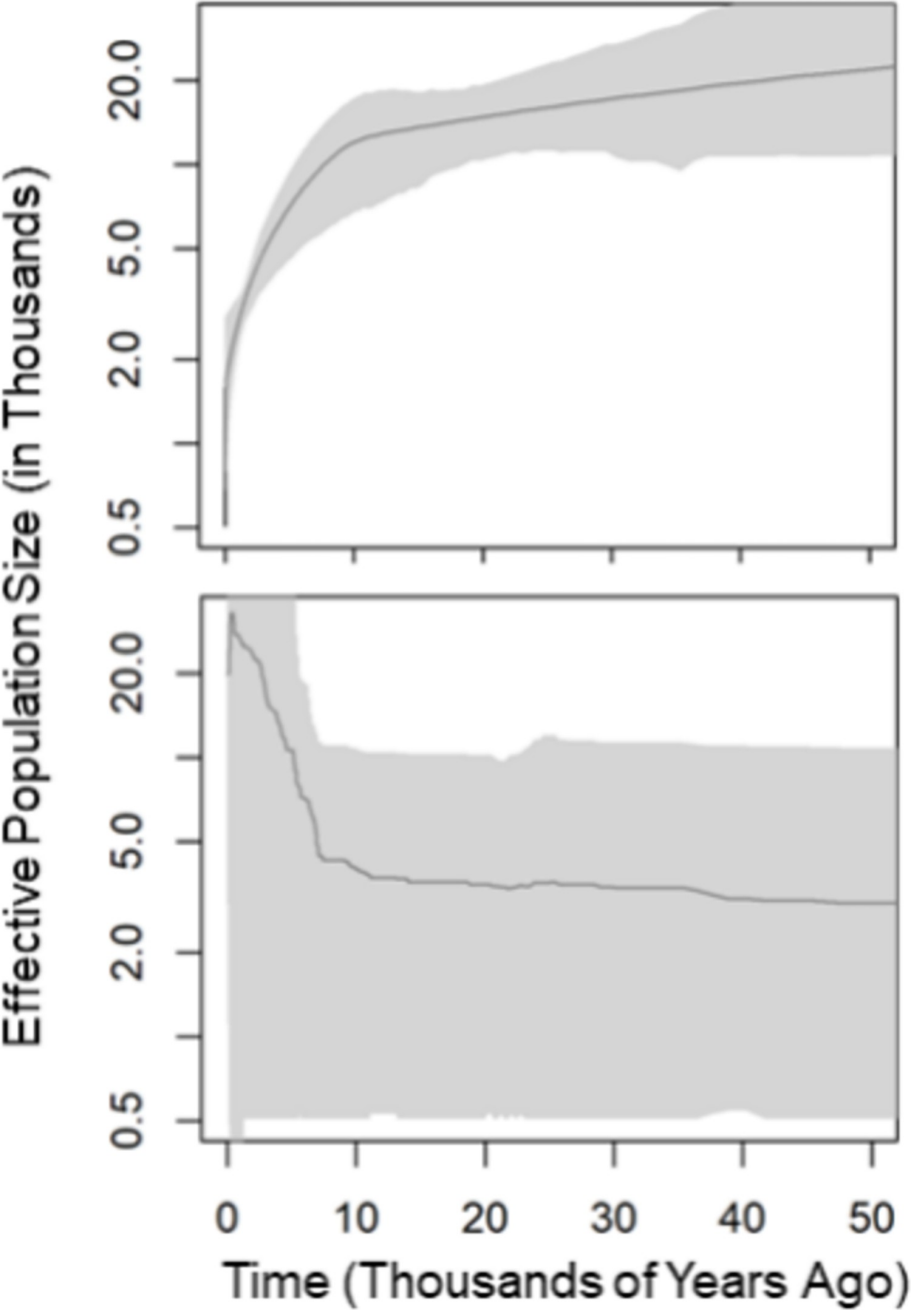
Extended Bayesian Skyline Plot for *Dipodomys elator* (top) from 34 individuals and 47 loci and for *D*. *ordii* (bottom) from 15 individuals and 49 loci. X-axis is thousands of years ago. Y-axis is effective population size (N_e_) in thousands. The y-axis is on a log scale. The dark line on each plot is the mean effective population size, while the shaded gray portions represent the upper highest posterior density (HPD) estimate and the lower highest posterior density (LHD).

The Extended Bayesian Skyline plot for *D*. *elator* was constructed with 24 individuals and 47 loci. The plot for *D*. *elator* showed a decline in effective population size over the last 10,000 years, to an approximate current N_e_ of 500. For *D*. *ordii*, we used 15 individuals and 49 loci. In this case, N_e_ has increased or remained stable in the last 5,000 years. The current value is estimated to stand at about 20,000 individuals (Fig 2).

### Population substructure

**Fig 3:**
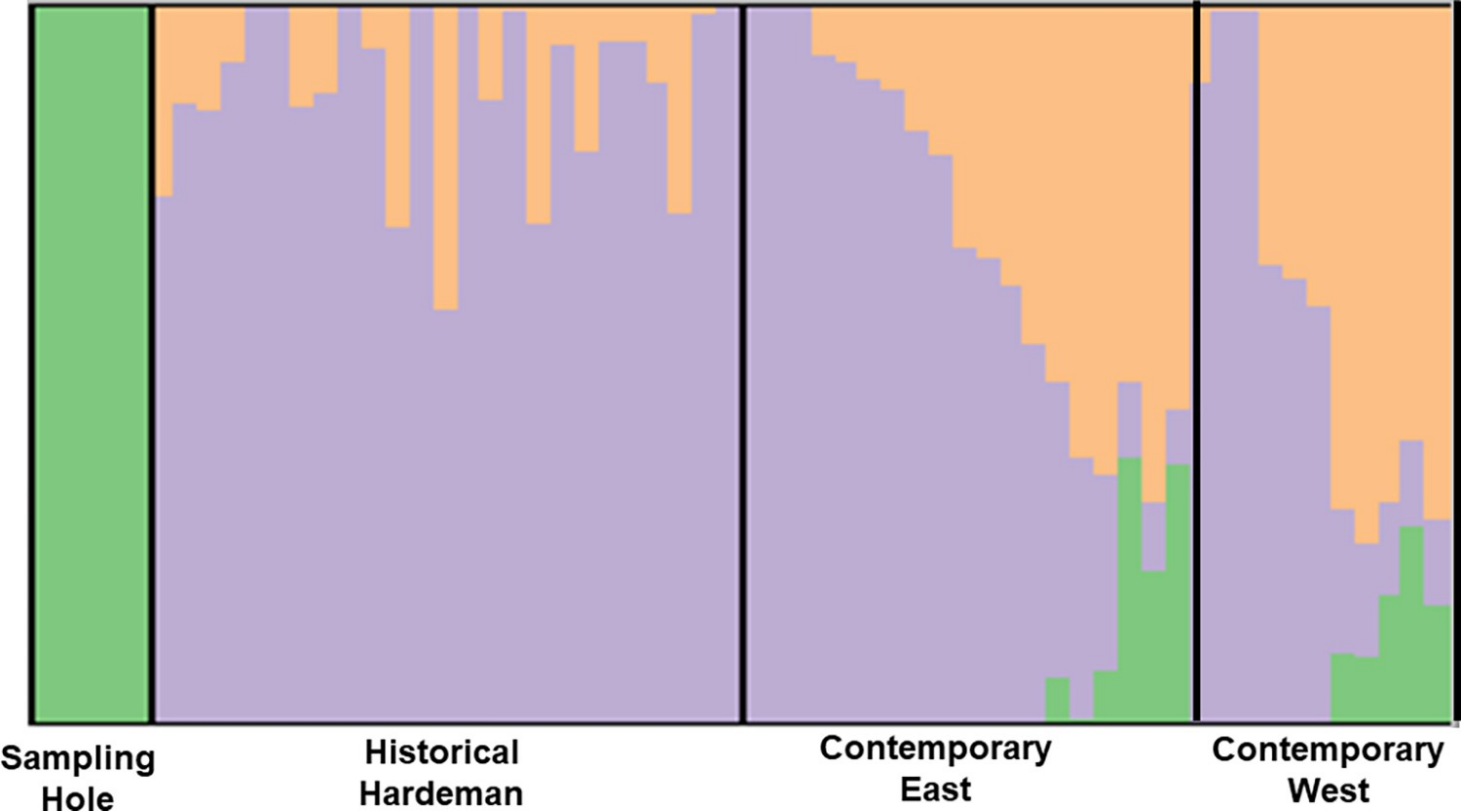
STRUCTURE plot of 60 *D*. *elator* samples across three time periods (see text for time breakdown). The “sampling gap” individuals, those that were found in the areas of Wilbarger and Baylor counties prior to 1980, are completely divergent from later samples;. These results show greater admixture among contemporary subpopulations, though there appears to be some structure in the contemporary east and west samples.

**Fig 4:**
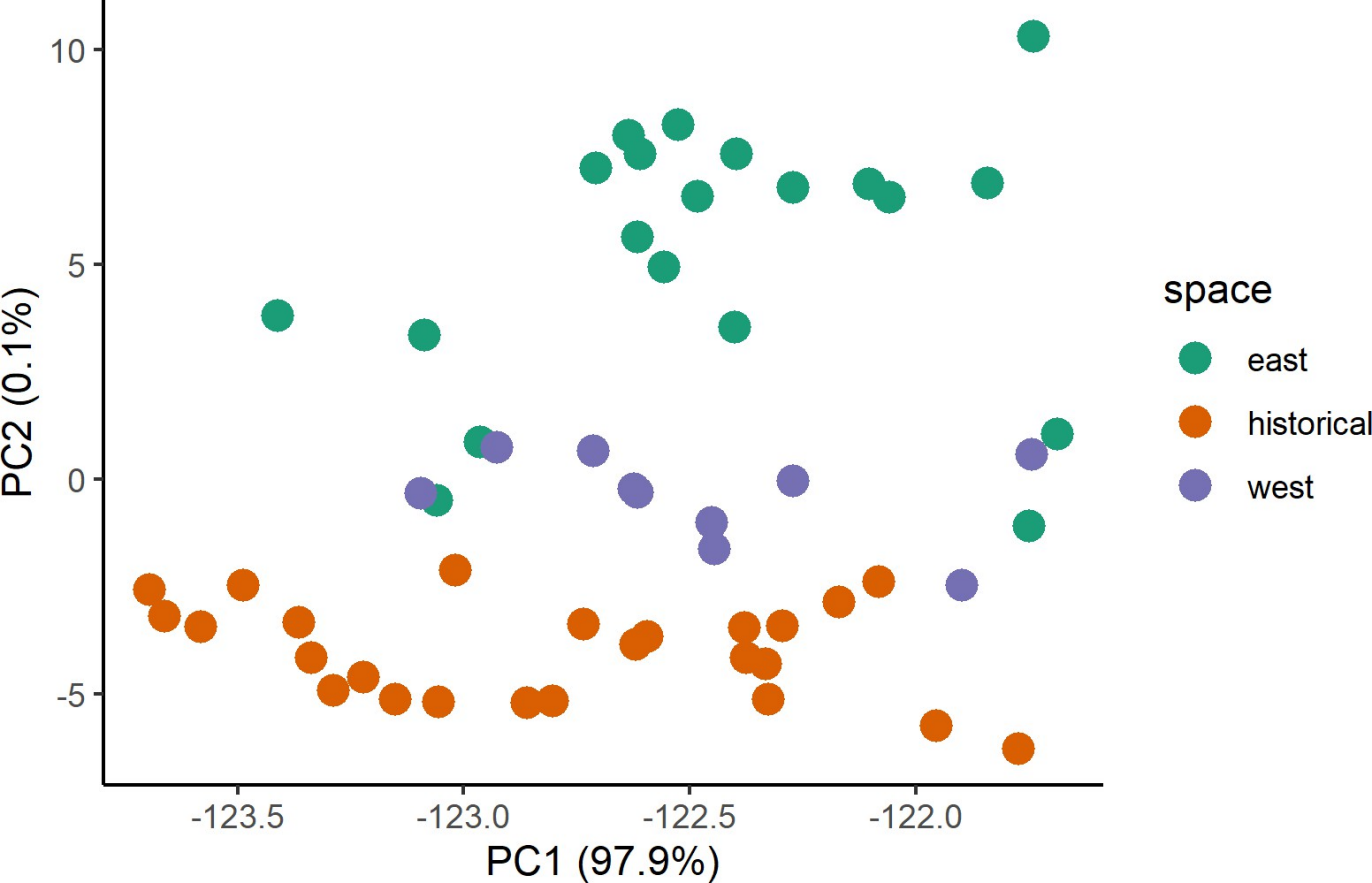
Principal components analysis on the genotypes for 55 *D*. *elator* samples (historical and contemporary) using the dudi.pca function in R package ‘adegenet’. While there are no clear clusters emerging on PC1, geographic location seems to correspond with PC2.

Based on log-likelihood scores (S4 Table) and their respective variances from STRUCTURE, the “best” value for the number of clusters present in the data for *D*. *elator* was 3 (Fig 3). For the PCA bi-plot, PC1 accounts for almost 98% of the variation found in the dataset and shows geographic separation along PC2, which only accounts for 0.1% of the variation (Fig 4). Most contemporary samples from the west cluster together and are nested within historical samples, based on Nei’s genetic distance (S1 Fig). The historical PCA for *D*. *elator* samples excluding those from the 1960s, which were removed due to poor data quality, shows that all individuals that were taken from the same region (Hardeman County) cluster near the origin and a difference in data quality could result in the three outlying samples (S2 Fig).

## Discussion

This study evaluated changes in genetic diversity across time and space by comparing a rare species with a hypothesized amorphous and restricted distribution to a more common congener with a larger, more defined range. This is only the third population genetic study on *Dipodomys elator* in over 30 years, and it is the first to make use of genomic techniques, screening from tens to thousands of markers across the genome, making the study valuable for current and future conservation efforts of *D*. *elator*. In Hamilton et al. 1987 [22], allozyme markers were used to conclude that there was moderate genetic differentiation among three *D*. *elator* localities (Hardeman, Wilbarger, and Wichita counties). This is seemingly incongruent with our results in which we observed little genetic differentiation (F_ST_ = 0.041), but the difference could simply be the result of the markers used (SNPs versus allozymes). Furthermore, lower heterozygosity in examined nuclear SNPs could reflect the low relative age or variation of the species as most SNPs are likely to be neutral. Similarly, bottleneck event(s) that may have occurred, could have potentially wiped arising genetic variation and homogenized standing populations. Such event was hypothesized in Pfau et al. 2019, but not supported (or detected) by either the estimations of N_e_ or the Extended Bayesian Skyline Plot method (Fig 2).

More recently, Pfau et al. (2019) [23] observed low mitochondrial DNA variation but high microsatellite diversity within the species and attributed this difference to mtDNA having lower effective population size than neutral nuclear markers such as microsatellites and RAD loci [61]. They concluded that genetic drift and not gene flow has had a greater impact on configuring *D*. *elator* genetic diversity, though this impact is minimal. Indeed, genetic drift could play a role in structuring mitochondrial DNA diversity, but more time would be needed to detect reduction of diversity in the nuclear genome using traditional markers such as microsatellites. An insufficient number of polymorphic microsatellite loci limits genetic resolution between individuals with supposed low population-level diversity. Our results suggest that RAD loci can provide greater resolution than with microsatellites when investigating populations with seemingly weak population structure [62].

Together, these three studies, using allozyme, mtDNA, microsatellite, and RAD-Seq markers, offer numerous mean geographic estimates of F_ST_ within this species. In Hamilton et al. 1987 [22], the mean F_ST_ was found to be 0.102, Pfau et al. 2019 [23] estimated F_ST_ of 0.096 from their late 1960s samples, and our study, at the greatest resolution of all previous studies, reveals a mean F_ST_ value of 0.041. Our lower mean value includes individuals sampled from localities not present in the previous two studies (i.e. Cottle and Childress counties). These results suggest low population differentiation corresponding with geography.

From an overall genetic diversity perspective, our data suggest that there has not been a substantial loss in genetic diversity over the last 30 years, despite what seems to be a decrease in the distribution (and possibly abundance) of *Dipodomys elator*, similar to what Statham et al. [63] found in *D*. *ingens*, the giant kangaroo rat. Like *D*. *elator*, the giant kangaroo rat is a species of conservation concern because habitat loss or change has reduced its historical range. So, despite a decline in distribution and census size, which is typically greater than N_e_, the genetic diversity of the species is sufficiently high to offset any short-term effects of inbreeding depression. This is supported by our NeEstimator and EBSP estimate of N_e_ of 170–500, though the NeEstimator value must also be taken under caution because many of the values were ‘Infinite’. This estimate exceeds the recommended minimum value to curtail inbreeding depression, as outlined by the 100/1000 rule [64]. Within this range, there exist enough individuals to mitigate fixation of deleterious alleles due to genetic drift but is low in the longer term (over thousands of years) as suggested by a steady decline in effective population size. In Pfau et al. 2019, it was also found that the N_e_ of this species was between 65 and 490 individuals.

Results from our contemporary samples confirm that subpopulation differentiation is not substantial (F_ST_ < 0.05). The STRUCTURE algorithm determined the best value of k to be 3, a finding also described by Pfau et al. 2019, who suggests weak genetic structure overall between east and west samples. The STRUCTURE plot indicates gene flow between hypothesized east and west demes, though it seems unclear exactly how many clusters the contemporary samples may represent. More clusters (i.e. subpopulations), while possible, does not seem parsimonious. Second, newly colonized subpopulations on the fringes of ranges can exhibit lower levels of genetic diversity than expected [65]. For our contemporary samples, this is not the case; the low mean value of F_ST_ (< 0.05) does not seem to support cluster sizes of k = 4 and k = 5.However, STRUCTURE, the PCA, and Nei’s genetic distances do not support two distinct subpopulations, suggesting there is a fair amount of gene flow in the region.

Our *a priori* subpopulations display low levels of inbreeding and very little genetic differentiation, suggesting one large interbreeding population, though not necessarily panmictic. Our samples were collected on opposite sides of a cline, separated by a region of inaccessible private land, so it was difficult to determine if the slight differentiation is due to geographic distance or if there is true population substructure and isolation from other habitat patches [66]. We included additional historic samples from specimens collected in the 1960s from areas within this “sampling gap” to answer this question. We anticipated that if the contemporary east and west subpopulations were indeed distinct, then genetic differentiation would be greater between them than to the samples from the sampling gap. In other words, a STRUCTURE plot would show the sampling gap samples as intermediate between the two. Alternatively, if the contemporary east and west subpopulations were considered one population then we would expect greater genetic differentiation between them and our “sampling gap” samples. Our results support the second prediction (Fig 3). However, the periods separating the datasets (anywhere between 20 and 50 years) and the relatively short generation times of kangaroo rats (about 3 years [55]) would lead to high genetic turnover, so these results must be interpreted with caution. If there is substantial genetic turnover, this too could indicate small current effective population size, which supports our estimate of 171–500. Though RAD-Seq and its various types rely on high quality, non-degraded DNA, we were fortunate to obtain enough data to investigate historical genetic diversity of *D*. *elator*.

As expected, our *D*. *ordii* samples exhibited higher genetic diversity estimates in nearly all categories despite our samples being collected from only five counties in north-central Texas. This emphasizes the substantial genetic diversity and evolutionary potential displayed by the more common *D*. *ordii*, compared with its much rarer congener. However, we were surprised to find that *D*. *ordii* had a greater inbreeding coefficient than *D*. *elator* across some analyses. This pattern can be attributed to sampling bias, given that we sampled from a small portion of the *D*. *ordii* range, and half of the *D*. *ordii* samples were collected from a single ranch in Dickens County, Texas. However, a relatedness test was performed, and no relatives were identified in the *D*. *ordii* set. This is possibly due to the poor data quality of the Dickens County samples (average read depth 1.4; S2 Table). Comparing between individuals from this ranch and a similarly situated subset of *D*. *elator* individuals, expected heterozygosity, π, and inbreeding coefficients were largely similar (S5 Table). This suggests that potentially related individuals of *D*. *elator* do not show reduced genetic diversity than similarly related *D*. *ordii* individuals.

With NeEstimator, we were unable to generate a value of N_e_ for the current sample of *D*. *ordii*, possibly because of the large effective population size of the species [67]. Instead, we used the value calculated from EBSP, which was approximately 20,000 individuals. The plot for *D*. *ordii* increased or remained stable, likely as a result of land use changes in the region and perhaps an indication of colonization of new habitat (northward) as glaciers receded after the Last Glacial Maximum 20,000 years ago [68, 69]. The conversion to cultivation, ranching activities, and cattle grazing in this region of Texas have been investigated as factors influencing *D*. *elator* populations [19]. The N_e_ of *D*. *elator* declined over time possibly due to land use changes, but this may be an oversimplification.

Coupled with low N_e_ estimates, and population surveys that recover or fail to locate *D*. *elator* in different localities, one possibility is that this population exhibits characteristics of a metapopulation [70, 71]. Metapopulation theory, the study of population dynamics, namely colonization and extinction, of smaller local populations that make up a larger population [71], has been discussed in the context of mammalian conservation biology because it accommodates populations in fragmented habitats [72], but empirical studies to develop metapopulation theory for threatened and endangered mammals are few (see [73, 74]). One reason for the difficulty to meet the original metapopulation criteria [75] is the stringency of the original criteria. In Elmhagen and Angerbjörn [76], the authors relaxed two criteria, adding that subpopulations, not the colonized habitat patch, are the discrete entity, and that these discrete subpopulations differ in their demography, implying asynchronicity. Based on field surveys, analysis of field notes, museum specimens, and species distribution models [21] there is evidence that the *D*. *elator* population may benefit from management consideration stemming from metapopulation theory.

However, because this connection to metapopulation theory is still tenuous, the overall population should still be monitored [77] and a long-term demographic study is warranted. Managing the metapopulation must be concerned with maintaining dispersal and gene flow among subpopulations, such as installing or improving habitat corridors or reintroducing *D*. *elator* to regions where the species appears extirpated. Should managers elect for more extreme measures to manage *D*. *elator* populations, such as captive breeding or population supplementation with the end goal of increasing genetic diversity, knowledge that the population is a metapopulation is critical if managers do not want to inadvertently remove local adaption. Lastly, it is important to note that the metapopulation in a conservation context has several assumptions. One assumption is the “equilibrium” between colonization and extinction across long time scales (i.e. if one patch goes extinct, another is colonized). This seems unlikely in many natural populations [78] but may be possible in *D*. *elator* despite its presumed low dispersal capabilities. This type of assumption can be used to appropriately model changes in demography and genetics of *D*. *elator*.

There is no lack of research on habitat associations, mainly those evaluating soil and vegetation changes, as they influence *D*. *elator*. These studies have greatly improved our understanding of this elusive rodent [16, 17, 19, 79, 80], but we still do not have answers to many basic biological questions. We do know, however, that the population of *D*. *elator* seems to track favorable habitat, albeit in a more restricted range than previously recorded [19].

Overall, the population of *D*. *elator* exhibits genetic variation lower than that of a species with a predictably greater effective population size. However, contemporary samples show no substantial decrease in genetic diversity from historical samples, suggesting that the *D*. *elator* population, though small and constantly shifting, has managed to maintain its genetic diversity.

This study demonstrates the effectiveness of using samples from gradations across the range, rather than at two extremes. Sampling from the extremes of a population range could lead researchers to inappropriate conclusions that could wrongly influence management decisions. Though the current effective population size of *D*. *elator* is estimated to be around 171 to 500 individuals, perhaps small population sizes are the status quo for this species. Increasing population size may be unsustainable for this species (greater competition, reduced resources, delayed or forgoing reproduction), but could come at the cost of reduced adaptive potential.

### Conservation implications

Researchers interested in natural genetic variation and population structure of mammals should consider the possibility that the population of their organisms of study could be exhibiting the characteristics of a metapopulation. This is especially important for species that are rarely seen or captured. Our findings suggest that the *D*. *elator* population could be a metapopulation that must be continuously monitored so that managers can detect immediately any significant losses in genetic diversity and evolutionary potential. Genomic data generated from this study can be used in future investigations. For example, data here can be used to represent another snapshot of the genetic diversity of the species, as the from historical samples served in this study. The data here can be used as a starting point to investigate adaptive loci within the species. Furthermore, given the current advances in molecular techniques and analyses, it is no longer necessary to limit samples in the temporal dimension. Doing so, especially for species that remain understudied, will prove detrimental to any long-term plan for management. We advise, whenever possible, the inclusion of historic and geographically represented samples to fully encapsulate temporal and spatial genetic variability within a possibly imperiled species.

## Supporting information

S1 Fig Nei’s genetic distance dendrogram for 55 *D*. *elator* samples (historical and contemporary). The patchy arrangement of individuals between contemporary spatial demes suggests gene flow between the hypothesized east and west populations, possibly indicative of a metapopulation.
(TIF)

S2 Fig Principal components analysis on 25 *D*. *elator* historical sample genotypes from Hardeman County using the dudi.pca function in R package ‘adegenet’. The three samples not clustering with all other is likely a result of different data quality.
(TIF)

S1 File Specimens examined section which includes coordinates for each sample used in the study. Some samples were found on private land, so those coordinates have been withheld.
(DOCX)

S2 File 3RAD protocol discussed in more detail. (DOCX)

S3 File Code used to run AfterQC and filtering steps in Stacks and r package *poppr*. (DOCX)

S4 File Principal components analysis for contemporary *D*. *elator* and *D*. *ordii* samples, shown separately. PC1 for the contemporary *D*. *elator* samples explained 9.9% of the variation, whereas PC2 accounted for an additional 5.7% of the variation. For historic *D*. *elator* samples, PC1 comprised of 6.9% of the variation, and PC2 explained 6.3% of the total variation. PC1 explained about 10% of the variation for contemporary *D*. *ordii* samples, and PC2 accounted for 9.8% of the total variation.
(XLSX)

S1 Table Seventy *Dipodomys elator* samples and 26 *D*. *ordii* samples used in the genetic analysis including temporal (historical (n = 33), contemporary (n = 37) subpopulations, spatial (east (n = 27) or west (n = 10)) subpopulation, the specific county the individual was found, tissue type, and the museum where the voucher was received. Museum codes are MSB (Museum of Southwestern Biology), MSU (Midwestern State University), and TTU (Texas Tech University).
(DOCX)

S2 Table Statistics on read depth and average number of reads that passed afterQC filtering for 70 *D*. elator samples and 26 *D*. ordii individuals. Sample indicated with asterisk were removed from downstream analyses due to missing data. Samples KR_01, KR_02, KR_04, KR_05, and KR_06 were not part of the initial sequencing but were added later. Raw read number has been included for these 5 samples.
(DOCX)

S3 Table Summary statistics calculated in Stacks for 26 *D*. *ordii* and 38 *D*. *elator* contemporary samples. Private alleles are those alleles not shared with any other subpopulation.
(DOCX)

S4 Table Log-likelihood and delta K values used in the Evanno method for *D*. *elator* STRUCTURE analysis. (DOCX)

S5 Table General summary statistics calculated in Stacks for a comparison between 3 individuals from each species that were collected in proximity (i.e. same tract of land). Private alleles are those alleles not shared with any other subpopulation. Observed and expected heterozygosity are the proportion of loci that are heterozygous based on Hardy-Weinberg frequencies. Historical observed and expected heterozygosity for *D*. *elator* are 0.042 and 0.038, respectively. π is a measure of nucleotide diversity. FIS indicates the inbreeding coefficient. The heterozygosity found in the Wichita samples is very large compared to what was found in the species as a whole. This is likely an artefact of which SNPs were used in this comparison. The SNPs evaluated are shared among both *D*. *elator* and *D*. *ordii*, and *D*. *ordii* is a common species, so the SNPs evaluated would be expected to have greater levels of diversity than SNPs evaluated in just *D*. *elator*.
(DOCX)
