# Supplementary figures and images for: Comparison of genetic variation between rare and common congeners of Dipodomys with estimates of contemporary and historical effective population size
Source: PLoS One. 2022 Sep 13;17(9):e0274554. doi: 10.1371/journal.pone.0274554 (PMC9469943; doi:10.1371/journal.pone.0274554)

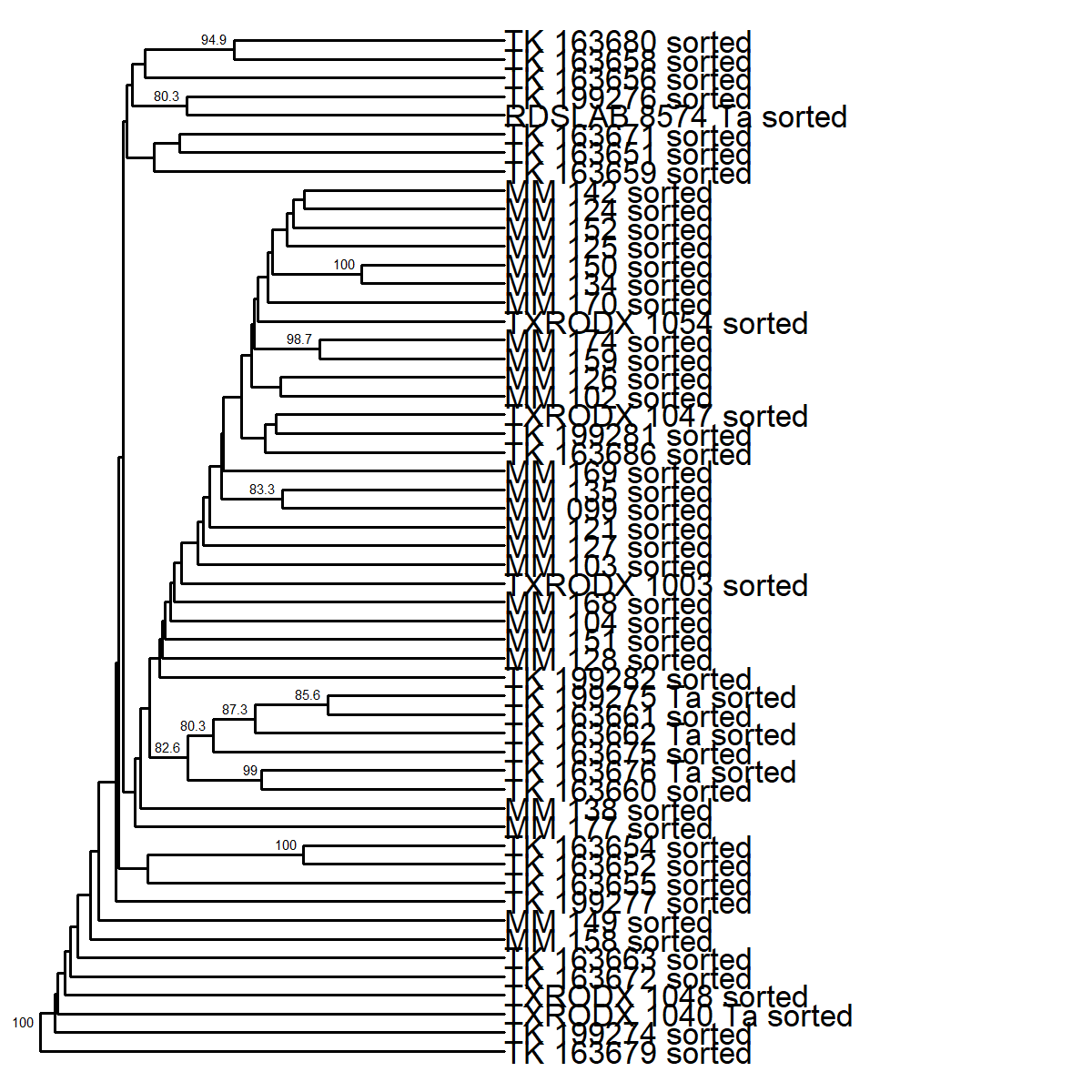

Supplement: S1 Fig — The patchy arrangement of individuals between contemporary spatial demes suggests gene flow between the hypothesized east and west populations, possibly indicative of a metapopulation. (TIF) [file pone.0274554.s001.tif]

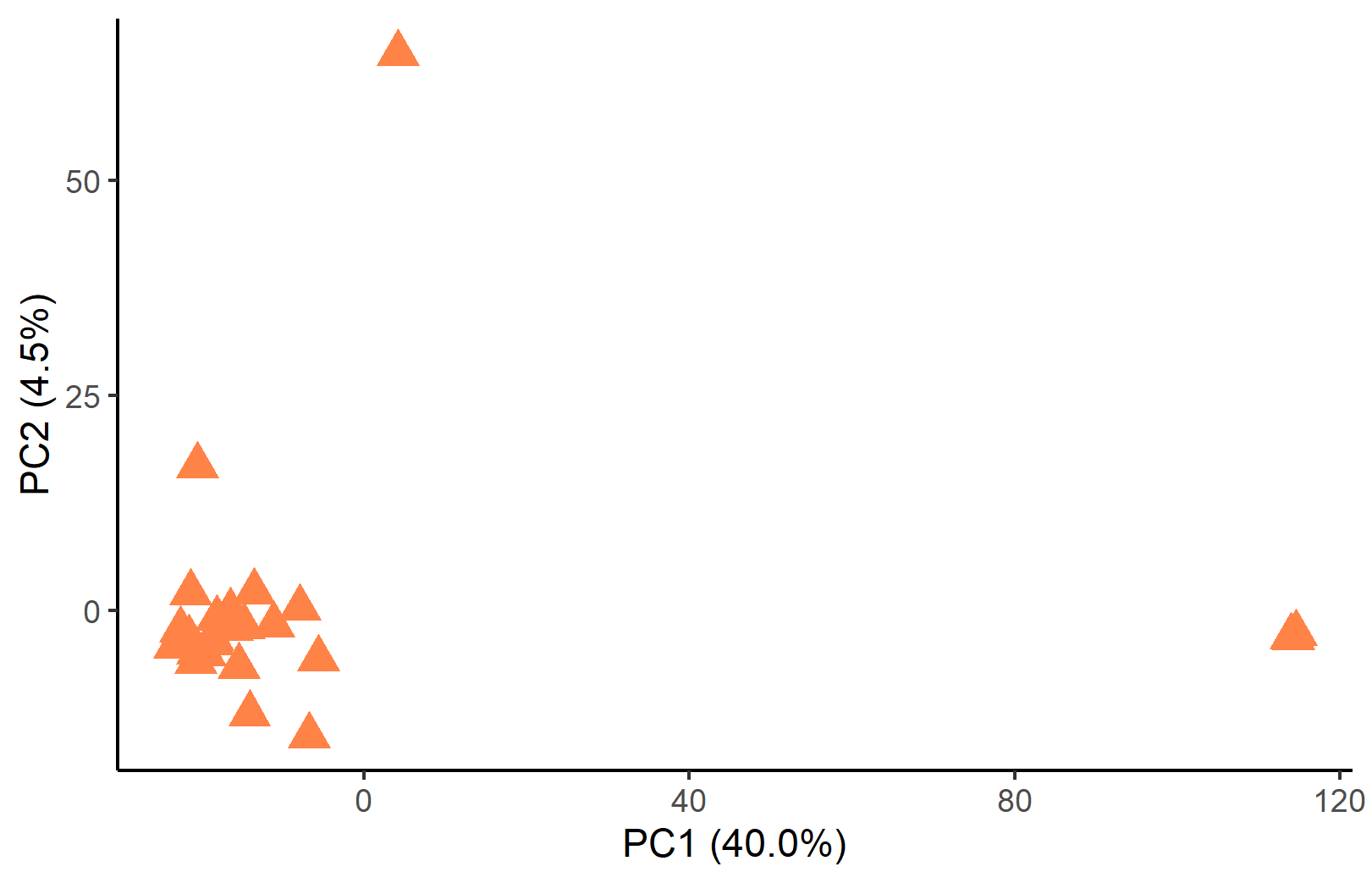

Supplement: S2 Fig — The three samples not clustering with all other is likely a result of different data quality. (TIF) [file pone.0274554.s002.tif]
