# Supplementary material for: Comparison of genetic variation between rare and common congeners of Dipodomys with estimates of contemporary and historical effective population size
Source: PLoS One. 2022 Sep 13;17(9):e0274554. doi: 10.1371/journal.pone.0274554 (PMC9469943; doi:10.1371/journal.pone.0274554)
Supplement: S1 File — Some samples were found on private land, so those coordinates have been withheld. (DOCX) [file pone.0274554.s003.docx]

Specimens whose DNA was extracted for this study are listed below according to the NSRL Tissue and Karyotype number (TK) or collector code. Individuals collected on private land do not have coordinates provided. Acronyms are as follows: M&M, Martin and Matocha samples; RDSLAB: R. D. Stevens Lab; TXRODX: Texas Rodent X series (collected by J. D. Stuhler and myself). Asterisks indicate the sample was not used in downstream analyses, unless indicated in manuscript text.

Specimens Examined –

*Dipodomy elator* (Historical)—USA: Texas; Hardeman, North Grid Station 1/6, 34.312679 N, 99.691390 W (M&M 099). USA: Texas; Hardeman County, South Grid Station 8/7, 34.310111 N, 99.689586 W (M&M 0101*). USA: Texas; Hardeman County, South Grid Station 1/8, 34.310335 N, 99.691180 W (M&M 0102). USA: Texas; Hardeman County, South Grid Station 7/5, 34.309753 N, 99.689817 W (M&M 0103). USA: Texas; Hardeman County, South Grid Station 8/3, 34.309389 N, 99.689607 W (M&M 0104). USA: Texas; Hardeman County, Station G-26, 34.303125 N, 99.684544 W (M&M 0121). USA: Texas; Hardeman County, Station G-24, 34.303299 N, 99.684828 W (M&M 0124). USA: Texas; Hardeman County, Station C-14, 34.304208 N, 99.689139 W (M&M 0125). USA: Texas; Hardeman County, Station C-15, 34.304370 N, 99.689124 W (M&M 0126). USA: Texas; Hardeman County, Station F-4, 34.307680 N, 99.697840 W (M&M 0127). USA: Texas; Hardeman County, Station F-11, 34.307735 N, 99.669199 W (M&M 0128). USA: Texas; Hardeman County, North Grid Station 7/8, 34.313719 N, 99.690839 W (M&M 0134). USA: Texas; Hardeman County, South Grid Station 3/8, 34.310321 N, 99.690707 W (M&M 0135). USA: Texas; Hardeman County, North Grid Station 8/8, 34.313895 N, 99.690824 W (M&M 0138). USA: Texas; Hardeman County, North Grid Station 1/3, 34.312725 N, 99.692059 W (M&M 0139*). USA: Texas; Hardeman County, North Grid Station 2/4, 34.312889 N, 99.691816 W (M&M 0140*). USA: Texas; Hardeman County, North Grid Station 2/3, 34.312905 N, 99.692039 W (M&M 0142). USA: Texas; Hardeman County, North Grid Station 8/8, 34.313895 N, 99.690824 W (M&M 0149). USA: Texas; Hardeman County, North Grid Station 8/5, 34.313946 N, 99.691477 W (M&M 0150). USA: Texas; Hardeman County, North Grid Station 7/8, 34.313719 N, 99.690839 W (M&M 0151). USA: Texas; Hardeman County, North Grid Station 8/8, 34.313895 N, 99.690824 W (M&M 0152). USA: Texas; Hardeman County, South Grid Station 8/4, 34.309565 N, 99.689598 W (M&M 0158). USA: Texas; Hardeman County, South Grid Station 1/1, 34.309059 N, 99.691191 W (M&M 0159). USA: Texas; Hardeman County, Station R-28, 34.303912 N, 99.689209 W (M&M 0168). USA: Texas; Hardeman County, North Grid Station 8/4, 34.313958 N, 99.691694 W (M&M 0169). USA: Texas; Hardeman County, Mil. 0.15, 34.301214 N, 99.689470 W (M&M 0170). USA: Texas; Hardeman County, South Grid Station 1/1, 34.309059 N, 99.691191 W (M&M 0174). USA: Texas; Hardeman County, North Grid Station 8/7, 34.313917 N, 99.691047 W (M&M 0177). Texas; Wilbarger County, 10 mi S, 5 M E Vernon, 34.0079 N, 99.1973 W, (MWSU 5855*), Texas; Baylor County, 15 mi NW Lake Kemp, 33.74360 N, 99.43929 W MWSU 8415*). Texas; Baylor County, 11 mi NE Lake Kemp, 33.70696 N, 99.13457 W (MWSU 8424*). Texas; Wilbarger County, Waggoner Ranch, 6 mi WSW Electra, 33.996 N, 99.015554 N (MSB 186201*, MSB 186203*).

*Dipodomys elator* (Contemporary)—USA: Texas; Wichita County, Wolf Rd, 34.1208 N, 98.93023 W (RDSLAB_8550*). USA: Texas; Wichita County, Preston Rd, 34.07283 N, -98.86272 W (RDSLAB_8563*). USA: Texas; Wichita County, Preston Rd, 34.07275 N, 98.85423 W (RDSLAB_8574_Ta). USA: Texas; Wichita County, Midway Church Rd, 34.04430 N, 98.87970 W(RDSLAB_8580*). USA: Texas; Wichita County, Hirschi Loop, 34.11943 N, 98.77187 W (RDSLAB_8557*). USA: Texas; Wichita County, Hirschi Loop, 34.07166 N, 98.46312 W (TK199281). USA: Texas; Wichita County, Van Low Rd, 34.05033 N, 98.69692 W (TK163651). USA; Wichita County, *private land*, (TK163652, TK163654, TK163655, TK163670*, TK163671, TK163672, TK163679, TK163680, TK163686). USA: Texas; Wichita County, Obenhaus Rd, 34.07120 N, 98.82848 W (TK199276). USA: Texas; Wilbarger County, Harrold Ln, 34.04168 N, 98.01659 W (TK199277). USA: Texas; Wilbarger County, Co Rd 97N, 34.21406 N, 99.33821 W (TXRODX_1040_Ta). USA: Texas; Wilbarger County, Co Rd 138E, 34.09517 N, 99.00391 W (TXRODX_1047). USA: Texas; Wilbarger County, Co Rd 114W, 34.23764 N, 99.33315 W (TXRODX_1049*). USA: Texas; Hardeman County, Biggs Rd, 34.39699 N, 99.93187 W (TK199282). USA: Texas; Hardeman County, Nippert Rd, 34.40655 N, 99.98540 W (TK163663). USA: Texas; Hardman County, Coburn Rd, 34.13356 N, 99.55254 W, (TXRODX_1048, TXRODX_1054). USA: Texas; Cottle County, Co Rd 408, 34.02980 N, 100.24430 W (TK163660). USA: Texas; Cottle County, Co Rd 411, 33.98935 N, 100.23994 W (TK163661). USA: Texas; Cottle County, Co Rd 403, 34.00385 N, 100.26154 W (TK163662_Ta, TK163675, TK163676_Ta). USA: Texas; Cottle County, Co Rd 403, 34.00381 N, 100.24001 (TK199274). USA: Texas; Childress County, Co Rd V, 34.45230 N, 100.13569 W (TXRODX_1003).

*Dipodomys ordii*—USA: Texas; Cottle County, Matador Wildlife Management Area, 34.14309 N, 100.35667 W (TK188084*), USA: Dickens County, 1 mile E Afton, 33.75180 N, 100.80011 W, (TK188126*, TK188128*, TK188131*, TK188132*, TK188133*, TK188134*, TK188135*, TK188140*, TK188141*). USA: Texas; Cottle County, CR 437, 33.91590 N, 100.20056 W (TK249564, TK249565). USA: Texas; Cottle County, CR 105, 34.01736 N, 100.36327 W (TK249566, TK249567). USA: Texas; Motley County, CR122, 34.21739 N, 101.00772 W (TK249581). USA: Texas: Childress County, CR 23, 34.48361 N, 100.03596 W (TK249582). USA: Texas: Childress County, CR 23, 34.45205 N, 100.03654 W (TK249583). USA: Texas: Childress County, CR I, 34.63069 N, 100.43504 W (TK249584, TK249585). USA: Texas: Childress County, CR I, 34.35508 N, 100.93720 W (TK249586). USA: Texas: Motley County, CR 121, 34.46447 N, 100.04136 W (TK249587*). USA: Texas: Childress County, CR V, 34.44963 N, 100.02817 W (TK249590). USA: Texas: Hall County, CR I, 34.63057 N, 100.46649 W (TK249591). USA: Texas: Hall County, CR 5, 34.34968 N, 100.35201 W (TK249592).
