# Supplementary material for: Comparison of genetic variation between rare and common congeners of Dipodomys with estimates of contemporary and historical effective population size
Source: PLoS One. 2022 Sep 13;17(9):e0274554. doi: 10.1371/journal.pone.0274554 (PMC9469943; doi:10.1371/journal.pone.0274554)
Supplement: S2 File — (DOCX) [file pone.0274554.s004.docx]

**Supplemental Methods 1**

RADseq libraries were prepared following the 3RAD protocol of Bayona-Vásquez et al. (2019). Five enzyme combinations were tested:

1. XbaI, EcoRI, and NheI,
2. MspI, BamHI, and ClaI,
3. XbaI, BamHI, and NheI,
4. MspI, EcoRI, and ClaI, and
5. NdeI, HindIII-HF, and CviQI.

Each enzyme combination test was performed in a total of 12 samples, 4 of these were liver samples from *Dipodomys ordii*, and the other 8 corresponded to *D. elator* samples from different sources (i.e., whisker, liver, toe, and genome amplification). Starting genomic DNA (gDNA) was normalized at 5 ng/µL, according to initial concentration values obtained with Qubit.

For testing enzyme combinations, gDNA was digested for 1 hr at 37 °C in a 15 µL reaction mix that consisted of: 1.5 µL 10x CutSmart Buffer, 1 µL of 5 µM double-stranded indexed iTru right adapter, 1 µL of 5 µM double-stranded indexed iTru left adapter, and 5 µL of gDNA, plus the necessary volumes to incorporate 10 U of each of the three enzymes, and reach the final volume with dH_2_O. After digestion, we added 2.75 µL dH_2_O, 1.5 µL ATP (10 µM), 0.5 µL 10x Ligase Buffer, and 0.25 µL T4 DNA Ligase (400 units/µL [NEB M0202L]) to each reaction. We incubated the digestion/adapter-ligation mixtures in a thermal cycler with the following conditions: 22°C for 20 min and 37°C for 10 min for two cycles followed by a single cycle of 80°C for 20 min. To remove remaining reagents and unincorporated adapters, we immediately added Sera-Mag Speedbeads (Thermo-Scientific, Waltham, MA, USA; see Glenn et al*.* 2019 for preparation methods) to each individual sample at a ratio of 1.2:1 Speedbeads to DNA, and we vortexed and incubated the mixture for 10 minutes at room temperature. Then, we cleaned-up samples using two washes of 80% EtOH, and we resuspended each in 20 µL of TE 1X.

To generate full-length library constructs, we combined 10 µL of each cleaned ligation product with 5.0 µL Kapa HiFi Buffer, 0.75 µL dNTPs at 10 µM, 3.75 µL dH_2_O, 0.5 µL Kapa HiFi DNA Polymerase at 1 U/µL, 2.5 µL iTru5 primer at 5 µM, and 2.5 µL iTru7 primer at 5 µM (Glenn et al. 2019). Each sample had an unique combination of iTru5 and iTru7 primers. The amplification was carried in a thermal cycler with the following thermal profile: 98°C for 2 min, followed by 15 cycles of 98°C for 20 sec, 60°C for 15 sec, 72°C for 30 sec; a final incubation at 72°C for 5 min; hold at 15°C. To validate that the library preparation process was successful, we ran 5 µL of PCR product with 2 µL loading dye on a 1.5% agarose gel for 45 minutes at 90 volts. A smear of DNA from ~300-800 bp, without a bandy pattern, indicated successful library preparation.

The library products of three enzyme combinations that presented the best digestion pattern according to the gels (i.e., 3, 4 and 5) were purified, pooled, size selected, and sequenced in an Illumina HiSeq 3000 to generate PE 150 data at Oklahoma Medical Research Foundation Genomics Core. Sequencing data was processed through Stacks v. 1.44 (Catchen et al., 2013), with default parameters. We found that the enzyme combo 4 (i.e., MspI/EcoRI/ClaI) obtained the highest number of raw reads for all samples, therefore the highest number of retained reads after filtering, and therefore the highest number of loci (both, fixed and polymorphic) assembled *de novo* for both species.

For the total samples, and based on the above results, the 4^th^ enzyme combo was used to prepare libraries for a total of 92 individuals. From these, gDNA from 48 samples was normalized at 20 ng/µL, and the rest were normalized at 3 ng/µL, according to initial concentration values obtained with Qubit. The library prep conditions were similar to the libraries above with some modifications outlined below.

To reach the final volume of 15 µL of the digestion mix, 5 µL of gDNA at 20 ng/µL were added to the mix, whereas 9.5 µL (and no dH_2_O) was added from gDNA at 3 ng/µL. Also, ligation products were pooled in equal volumes (i.e., 5 µL per sample) based on initial normalization values (i.e., one pool for samples starting with 20 ng/µL, and one pool for samples starting with 3 ng/µL). These pooled products were cleaned at a ratio of 1.25:1 Speedbeads to DNA, vortexed, and incubated for 10 minutes at room temperature. Then, we cleaned-up each pool using two washes of 80% EtOH, and resuspended each in 30 µL of TE 1X.

For PCR, the molecular ID tag protocol (Hoffberg et al., 2016; Bayona-Vásquez et al., 2019) to detect PCR duplicates was followed. First, three replicates of a one-cycle PCR were run per pool. Each replicate contained, 5.0 µL Kapa HiFi Buffer, 1.5 µL dNTPs at 10 µM, 22.5 µL dH_2_O, 1.0 µL Kapa HiFi DNA Polymerase at 1 U/µL, 5 µL iTru5-8N primer at 5 µM, and 10 µL of cleaned ligation product. The one-cycle conditions were: 98 °C for 60 sec, 60°C for 30 sec, 72°C for 60 sec, and incubation at 72°C for 5 min. The three replicates for each pool were joined and purified with a 1.7:1 Speedbeads to DNA ratio, vortexed, and incubuted at room temperature for 10 minutes. Then, each pool was washed with 80% EtOH, and resuspended in 22 µL of TE 1X.

Second, a limited-cycle PCR was performed in duplicate per pool. Each duplicate contained, 10.0 µL Kapa HiFi Buffer, 1.5 µL dNTPs at 10 µM, 17.5 µL dH_2_O, 1.0 µL Kapa HiFi DNA Polymerase at 1 U/µL, 5 µL iTru7 primer at 5 µM, 5 µL of P5 primer at 5 µM, and 10 µL of pooled and cleaned one-cycle product. The limited-cycle conditions were: 98 °C for 2 minutes; then 6 cycles of 98 °C for 20 sec, 60°C for 15 sec, 72°C for 30 sec, and incubation at 72°C for 5 min. After PCR, all replicates from both pools were pooled in one master pool and this was purified at a 1.5:1 Speedbeads to DNA ratio, cleaned as usual, and resuspended in 50 µL of TE 1X.

We quantified the master pool using Qubit Fluorometry (Life Technologies, Inc.), and size-selected pooled libraries using a Pippin Prep (Sage Science, Beverly, MA) with a 1.5% dye-free agarose gel cassette (CDF1510) and marker K set to capture fragments at 550 bp +/- 15%. Then the collected pool was cleaned with Speedbeads in a 2:1 ratio (Speedbead:DNA) and eluted in 1X TE buffer. Libraries were sequenced using an Illumina HiSeq 3000 to generate PE150 data at Oklahoma Medical Research Foundation Genomics Core.

Ten extra samples were prepared following the methods described for the samples used for testing the enzymes. In short, these were digested with the 4^th^ enzyme combination, ligated, and then amplified independently using 15 to 21 cycles during PCR, without performing the molecular ID tag protocol. All other steps, including size selection were performed as described above. These libraries were sequenced in an Illumina HiSeq 4000 to generate PE150 data at Novogene Inc.
