# Supplementary material for: Comparison of genetic variation between rare and common congeners of Dipodomys with estimates of contemporary and historical effective population size
Source: PLoS One. 2022 Sep 13;17(9):e0274554. doi: 10.1371/journal.pone.0274554 (PMC9469943; doi:10.1371/journal.pone.0274554)
Supplement: S3 File — (DOCX) [file pone.0274554.s005.docx]

Codes used for filtering

#AfterQC

#Where R1 and R2 are read 1 and read 2 of each sample

python after.py -1 R1.fq -2 R2.fq

#STACKS

#SNP calling was using after the gstacks program was run, using the following ‘populations’ code. This example is used for the contemporary *D. elator* samples and an -r flag of 0.75.

populations -P ../temporal/ -O ./2020/elator/east_west -M ./currentEL.txt -r 0.75 --hwe --structure --vcf --write_random_snp --ordered_export --genepop –fstats

#Additional filtering using *poppr* package in R. Genepop file was read into R as a genind object

obj_krat_ew <- read.genepop(file, ncode =2)

#Removes samples that have greater than 20% missing data

obj_krat_ewfil <- missingno(obj_krat_ew, type= "loci", cutoff = 0.20)
