# Supplementary material for: Comparison of genetic variation between rare and common congeners of Dipodomys with estimates of contemporary and historical effective population size
Source: PLoS One. 2022 Sep 13;17(9):e0274554. doi: 10.1371/journal.pone.0274554 (PMC9469943; doi:10.1371/journal.pone.0274554)
Supplement: S1 Table — Museum codes are MSB (Museum of Southwestern Biology), MSU (Midwestern State University), and TTU (Texas Tech University). (DOCX) [file pone.0274554.s007.docx]

| Sample Name | Species | Temporal Deme | Spatial Deme | County | Tissue | Museum |
| --- | --- | --- | --- | --- | --- | --- |
| MM_099 | *D. elator* | Historical | West | Hardeman | toe |  |
| MM_101 | *D. elator* | Historical | West | Hardeman | toe |  |
| MM_102 | *D. elator* | Historical | West | Hardeman | toe |  |
| MM_103 | *D. elator* | Historical | West | Hardeman | toe |  |
| MM_104 | *D. elator* | Historical | West | Hardeman | toe |  |
| MM_121 | *D. elator* | Historical | West | Hardeman | toe |  |
| MM_124 | *D. elator* | Historical | West | Hardeman | toe |  |
| MM_125 | *D. elator* | Historical | West | Hardeman | toe |  |
| MM_126 | *D. elator* | Historical | West | Hardeman | toe |  |
| MM_127 | *D. elator* | Historical | West | Hardeman | toe |  |
| MM_128 | *D. elator* | Historical | West | Hardeman | toe |  |
| MM_134 | *D. elator* | Historical | West | Hardeman | toe |  |
| MM_135 | *D. elator* | Historical | West | Hardeman | toe |  |
| MM_138 | *D. elator* | Historical | West | Hardeman | toe |  |
| MM_139 | *D. elator* | Historical | West | Hardeman | toe |  |
| MM_140 | *D. elator* | Historical | West | Hardeman | toe |  |
| MM_142 | *D. elator* | Historical | West | Hardeman | toe |  |
| MM_149 | *D. elator* | Historical | West | Hardeman | toe |  |
| MM_150 | *D. elator* | Historical | West | Hardeman | toe |  |
| MM_151 | *D. elator* | Historical | West | Hardeman | toe |  |
| MM_152 | *D. elator* | Historical | West | Hardeman | toe |  |
| MM_158 | *D. elator* | Historical | West | Hardeman | toe |  |
| MM_159 | *D. elator* | Historical | West | Hardeman | toe |  |
| MM_168 | *D. elator* | Historical | West | Hardeman | toe |  |
| MM_169 | *D. elator* | Historical | West | Hardeman | toe |  |
| MM_170 | *D. elator* | Historical | West | Hardeman | toe |  |
| MM_174 | *D. elator* | Historical | West | Hardeman | toe |  |
| MM_177 | *D. elator* | Historical | West | Hardeman | toe |  |
| KR_01 | *D. elator* | Historical | East | Wilbarger | toe | MSB |
| KR_03 | *D. elator* | Historical | East | Wilbarger | toe | MSB |
| KR_04 | *D. elator* | Historical | East | Wilbarger | toe | MSB |
| KR_05 | *D. elator* | Historical | East | Baylor | toe | MWS |
| KR_06 | *D. elator* | Historical | East | Baylor | toe | MWS |
| RDSLAB_8510 | *D. elator* | Contemporary | East | Wichita | whisker | TTU |
| RDSLAB_8550 | *D. elator* | Contemporary | East | Wichita | whisker | TTU |
| RDSLAB_8557 | *D. elator* | Contemporary | East | Wichita | whisker | TTU |
| RDSLAB_8563 | *D. elator* | Contemporary | East | Wichita | whisker | TTU |
| RDSLAB_8574_Ta | *D. elator* | Contemporary | East | Wichita | tail | TTU |
| RDSLAB_8580 | *D. elator* | Contemporary | East | Wichita | whisker | TTU |
| TK_163651 | *D. elator* | Contemporary | East | Wichita | whisker | TTU |
| TK_163652 | *D. elator* | Contemporary | East | Wichita | whisker | TTU |
| TK_163654 | *D. elator* | Contemporary | East | Wichita | whisker | TTU |
| TK_163655 | *D. elator* | Contemporary | East | Wichita | whisker | TTU |
| TK_163656 | *D. elator* | Contemporary | East | Wichita | whisker | TTU |
| TK_163658 | *D. elator* | Contemporary | East | Wichita | whisker | TTU |
| TK_163659 | *D. elator* | Contemporary | East | Wichita | whisker | TTU |
| TK_163670 | *D. elator* | Contemporary | East | Wichita | whisker | TTU |
| TK_163671 | *D. elator* | Contemporary | East | Wichita | whisker | TTU |
| TK_163672 | *D. elator* | Contemporary | East | Wichita | whisker | TTU |
| TK_163679 | *D. elator* | Contemporary | East | Wichita | whisker | TTU |
| TK_163680 | *D. elator* | Contemporary | East | Wichita | whisker | TTU |
| TK_163686 | *D. elator* | Contemporary | East | Wichita | whisker | TTU |
| TK_199281 | *D. elator* | Contemporary | East | Wichita | liver | TTU |
| TK_163660 | *D. elator* | Contemporary | West | Cottle | whisker | TTU |
| TK_163661 | *D. elator* | Contemporary | West | Cottle | whisker | TTU |
| TK_163662_Ta | *D. elator* | Contemporary | West | Cottle | tail | TTU |
| TK_163675 | *D. elator* | Contemporary | West | Cottle | whisker | TTU |
| TK_163676_Ta | *D. elator* | Contemporary | West | Cottle | tail | TTU |
| TK_199274 | *D. elator* | Contemporary | West | Cottle | whisker | TTU |
| TK_199275_Ta | *D. elator* | Contemporary | West | Cottle | tail | TTU |
| TK_163663 | *D. elator* | Contemporary | West | Hardeman | whisker | TTU |
| TK_199282 | *D. elator* | Contemporary | West | Hardeman | liver | TTU |
| TXRODX_1003 | *D. elator* | Contemporary | West | Childress | buccal | TTU |
| TK_199276 | *D. elator* | Contemporary | East | Wilbarger | liver | TTU |
| TK_199277 | *D. elator* | Contemporary | East | Wilbarger | liver | TTU |
| TXRODX_1040_Ta | *D. elator* | Contemporary | East | Wilbarger | tail | TTU |
| TXRODX_1047 | *D. elator* | Contemporary | East | Wilbarger | whisker | TTU |
| TXRODX_1048 | *D. elator* | Contemporary | East | Wilbarger | whisker | TTU |
| TXRODX_1049 | *D. elator* | Contemporary | East | Wilbarger | whisker | TTU |
| TXRODX_1054 | *D. elator* | Contemporary | East | Wilbarger | whisker | TTU |
| TK_187667 | *D. ordii* | Contemporary |  | Dickens | whisker | TTU |
| TK_188084 | *D. ordii* | Contemporary |  | Dickens | whisker | TTU |
| TK_188088 | *D. ordii* | Contemporary |  | Dickens | whisker | TTU |
| TK_188126 | *D. ordii* | Contemporary |  | Dickens | whisker | TTU |
| TK_188128 | *D. ordii* | Contemporary |  | Dickens | whisker | TTU |
| TK_188131 | *D. ordii* | Contemporary |  | Dickens | whisker | TTU |
| TK_188132 | *D. ordii* | Contemporary |  | Dickens | whisker | TTU |
| TK_188133 | *D. ordii* | Contemporary |  | Dickens | whisker | TTU |
| TK_188134 | *D. ordii* | Contemporary |  | Dickens | whisker | TTU |
| TK_188135 | *D. ordii* | Contemporary |  | Dickens | whisker | TTU |
| TK_188140 | *D. ordii* | Contemporary |  | Dickens | whisker | TTU |
| TK_188141 | *D. ordii* | Contemporary |  | Dickens | whisker | TTU |
| TK_249564 | *D. ordii* | Contemporary |  | Cottle | liver | TTU |
| TK_249565 | *D. ordii* | Contemporary |  | Cottle | liver | TTU |
| TK_249566 | *D. ordii* | Contemporary |  | Cottle | liver | TTU |
| TK_249567 | *D. ordii* | Contemporary |  | Cottle | liver | TTU |
| TK_249581 | *D. ordii* | Contemporary |  | Motley | liver | TTU |
| TK_249582 | *D. ordii* | Contemporary |  | Childress | liver | TTU |
| TK_249583 | *D. ordii* | Contemporary |  | Childress | liver | TTU |
| TK_249584 | *D. ordii* | Contemporary |  | Hall | liver | TTU |
| TK_249585 | *D. ordii* | Contemporary |  | Hall | liver | TTU |
| TK_249586 | *D. ordii* | Contemporary |  | Hall | liver | TTU |
| TK_249587 | *D. ordii* | Contemporary |  | Motley | liver | TTU |
| TK_249590 | *D. ordii* | Contemporary |  | Childress | liver | TTU |
| TK_249591 | *D. ordii* | Contemporary |  | Hall | liver | TTU |
| TK_249592 | *D. ordii* | Contemporary |  | Childress | liver | TTU |
