# Supplementary material for: Comparison of genetic variation between rare and common congeners of Dipodomys with estimates of contemporary and historical effective population size
Source: PLoS One. 2022 Sep 13;17(9):e0274554. doi: 10.1371/journal.pone.0274554 (PMC9469943; doi:10.1371/journal.pone.0274554)
Supplement: S2 Table — Sample indicated with asterisk were removed from downstream analyses due to missing data. Samples KR_01, KR_02, KR_04, KR_05, and KR_06 were not part of the initial sequencing but were added later. Raw read number has been included for these 5 samples. (DOCX) [file pone.0274554.s008.docx]

| Sample | Tissue | Average Reads | Read_Depth | Temporal | Species |
| --- | --- | --- | --- | --- | --- |
| MM_099 | toe | 395127.5 | 7.18239 | Historical | *elator* |
| MM_101* | toe | 20454 | 1.39479 | Historical | *elator* |
| MM_102 | toe | 1298797 | 17.8506 | Historical | *elator* |
| MM_103 | toe | 503176 | 9.20962 | Historical | *elator* |
| MM_104 | toe | 512776 | 9.33275 | Historical | *elator* |
| MM_121 | toe | 581413 | 10.1464 | Historical | *elator* |
| MM_124 | toe | 2532246.5 | 43.9169 | Historical | *elator* |
| MM_125 | toe | 1104165 | 19.3349 | Historical | *elator* |
| MM_126 | toe | 1043071 | 18.0734 | Historical | *elator* |
| MM_127 | toe | 472124 | 8.58382 | Historical | *elator* |
| MM_128 | toe | 540606.5 | 9.66167 | Historical | *elator* |
| MM_134 | toe | 1228470 | 20.8201 | Historical | *elator* |
| MM_135 | toe | 628231 | 11.1149 | Historical | *elator* |
| MM_138 | toe | 325040 | 6.27427 | Historical | *elator* |
| MM_139* | toe | 947914 | 9 | Historical | *elator* |
| MM_140* | toe | 314129 | 1.93827 | Historical | *elator* |
| MM_142 | toe | 1423724 | 24.6774 | Historical | *elator* |
| MM_149 | toe | 301859 | 5.7875 | Historical | *elator* |
| MM_150 | toe | 923783.5 | 16.0203 | Historical | *elator* |
| MM_151 | toe | 469553 | 8.62434 | Historical | *elator* |
| MM_152 | toe | 782656.5 | 13.8555 | Historical | *elator* |
| MM_158 | toe | 353380.5 | 6.66937 | Historical | *elator* |
| MM_159 | toe | 634231 | 11.3861 | Historical | *elator* |
| MM_168 | toe | 433411.5 | 7.84678 | Historical | *elator* |
| MM_169 | toe | 659931.5 | 11.5532 | Historical | *elator* |
| MM_170 | toe | 705962.5 | 12.6145 | Historical | *elator* |
| MM_174 | toe | 579249.5 | 10.2103 | Historical | *elator* |
| MM_177 | toe | 405961 | 7.27795 | Historical | *elator* |
| KR_01* | toe | 2904051 | 112.338 | Historical | *elator* |
| KR_02* | toe | 1447248 | 89.726 | Historical | *elator* |
| KR_04* | toe | 2401398 | 4.53125 | Historical | *elator* |
| KR_05* | toe | 2812680 | 116.319 | Historical | *elator* |
| KR_06* | toe | 302434 | 19.2075 | Historical | *elator* |
| RDSLAB_8510* | whisker | 2623.5 | 1.37864 | Contemporary | *elator* |
| RDSLAB_8550* | whisker | 12805.5 | 1.56647 | Contemporary | *elator* |
| RDSLAB_8557* | whisker | 24409 | 1.68138 | Contemporary | *elator* |
| RDSLAB_8563* | whisker | 62946 | 2.25458 | Contemporary | *elator* |
| RDSLAB_8574_Ta | tail | 620333.5 | 14.2247 | Contemporary | *elator* |
| RDSLAB_8580* | whisker | 15868 | 1.59255 | Contemporary | *elator* |
| TK_163651 | whisker | 631757 | 15.4234 | Contemporary | *elator* |
| TK_163652 | whisker | 599376.5 | 14.5793 | Contemporary | *elator* |
| TK_163654 | whisker | 458598.5 | 11.0379 | Contemporary | *elator* |
| TK_163655 | whisker | 375775 | 9.07129 | Contemporary | *elator* |
| TK_163656 | whisker | 344943 | 8.73507 | Contemporary | *elator* |
| TK_163658 | whisker | 576695 | 14.0397 | Contemporary | *elator* |
| TK_163659 | whisker | 350330.5 | 8.60205 | Contemporary | *elator* |
| TK_163670* | whisker | 58163 | 2.19992 | Contemporary | *elator* |
| TK_163671 | whisker | 682759.5 | 16.4955 | Contemporary | *elator* |
| TK_163672 | whisker | 177529 | 4.46134 | Contemporary | *elator* |
| TK_163679 | whisker | 302208 | 7.26722 | Contemporary | *elator* |
| TK_163680 | whisker | 494824 | 11.2533 | Contemporary | *elator* |
| TK_163686 | whisker | 104079 | 3.04785 | Contemporary | *elator* |
| TK_199281 | liver | 1866981.5 | 64.714 | Contemporary | *elator* |
| TK_163660 | whisker | 122562.5 | 3.41006 | Contemporary | *elator* |
| TK_163661 | whisker | 154481 | 4.07298 | Contemporary | *elator* |
| TK_163662_Ta | tail | 852167.5 | 20.9922 | Contemporary | *elator* |
| TK_163675 | whisker | 113102.5 | 3.3269 | Contemporary | *elator* |
| TK_163676_Ta | tail | 150233.5 | 3.89684 | Contemporary | *elator* |
| TK_199274 | liver | 343528 | 7.87905 | Contemporary | *elator* |
| TK_199275_Ta | tail | 895835 | 21.6236 | Contemporary | *elator* |
| TK_163663 | whisker | 370024 | 8.99071 | Contemporary | *elator* |
| TK_199282 | liver | 356185 | 8.344 | Contemporary | *elator* |
| TXRODX_1003 | buccal | 123756.5 | 3.02503 | Contemporary | *elator* |
| TK_199276 | liver | 908920.5 | 20.9404 | Contemporary | *elator* |
| TK_199277 | liver | 1066750 | 24.3715 | Contemporary | *elator* |
| TXRODX_1040_Ta | tail | 269530 | 5.81045 | Contemporary | *elator* |
| TXRODX_1047 | whisker | 90635.5 | 2.82761 | Contemporary | *elator* |
| TXRODX_1048 | whisker | 251642 | 6.19019 | Contemporary | *elator* |
| TXRODX_1049* | whisker | 43833 | 1.96466 | Contemporary | *elator* |
| TXRODX_1054 | whisker | 86681 | 2.68426 | Contemporary | *elator* |
| TK_249564_sorted | liver | 354149 | 6.12017 | Contemporary | *ordii* |
| TK_249565_sorted | liver | 1421011 | 22.7736 | Contemporary | *ordii* |
| TK_249566_sorted | liver | 1540983.5 | 23.1302 | Contemporary | *ordii* |
| TK_249567_sorted | liver | 1236801.5 | 20.28 | Contemporary | *ordii* |
| TK_249581_sorted | liver | 665028 | 11.1909 | Contemporary | *ordii* |
| TK_249582_sorted | liver | 315838 | 5.5297 | Contemporary | *ordii* |
| TK_249583_sorted | liver | 318959.5 | 5.28007 | Contemporary | *ordii* |
| TK_249584_sorted | liver | 798974 | 14.3977 | Contemporary | *ordii* |
| TK_249585_sorted | liver | 592890.5 | 9.69508 | Contemporary | *ordii* |
| TK_249586_sorted | liver | 777079.5 | 13.53 | Contemporary | *ordii* |
| TK_249587_sorted* | liver | 167917 | 3.26315 | Contemporary | *ordii* |
| TK_249590_sorted | liver | 320213.5 | 4.90785 | Contemporary | *ordii* |
| TK_249591_sorted | liver | 470121.5 | 7.89809 | Contemporary | *ordii* |
| TK_249592_sorted | liver | 291624 | 4.83286 | Contemporary | *ordii* |
| TK_187667_sorted* | whisker | 819.5 | 0 | Contemporary | *ordii* |
| TK_188084_sorted* | whisker | 1791 | 1.2803 | Contemporary | *ordii* |
| TK_188088_sorted* | whisker | 6120 | 0 | Contemporary | *ordii* |
| TK_188126_sorted* | whisker | 2514.5 | 1.32209 | Contemporary | *ordii* |
| TK_188128_sorted* | whisker | 97172 | 2.51069 | Contemporary | *ordii* |
| TK_188131_sorted* | whisker | 1693.5 | 1.34225 | Contemporary | *ordii* |
| TK_188132_sorted* | whisker | 381 | 1.08333 | Contemporary | *ordii* |
| TK_188133_sorted* | whisker | 329 | 1.25 | Contemporary | *ordii* |
| TK_188134_sorted* | whisker | 5566 | 1.37255 | Contemporary | *ordii* |
| TK_188135_sorted* | whisker | 2348 | 1.32886 | Contemporary | *ordii* |
| TK_188140_sorted* | whisker | 13049 | 1.50535 | Contemporary | *ordii* |
| TK_188141_sorted* | whisker | 1989.5 | 1.31757 | Contemporary | *ordii* |
