# Supplementary material for: Comparison of genetic variation between rare and common congeners of Dipodomys with estimates of contemporary and historical effective population size
Source: PLoS One. 2022 Sep 13;17(9):e0274554. doi: 10.1371/journal.pone.0274554 (PMC9469943; doi:10.1371/journal.pone.0274554)
Supplement: S3 Table — Private alleles are those alleles not shared with any other subpopulation. (DOCX) [file pone.0274554.s009.docx]

| Missingness value  (-r) | Analyzed SNPs | Group | Private alleles |
| --- | --- | --- | --- |
| 0.95 | 7 | *D. ordii* | 0 |
|  |  | *D.elator (*Contemporary) | 0 |
| 0.75 | 4,767 | *D.ordii* | 1 |
|  |  | *D. elator (*Contemporary) | 0 |
| 0.5 | 31,068 | *D.ordii* | 2,574 |
|  |  | *D. elator (*Contemporary) | 1,134 |
| 0.25 | 61,208 | *D. ordii* | 5,288 |
|  |  | *D. elator (*Contemporary) | 2,318 |

**Table S3. Summary statistics calculated in Stacks for 26 *D. ordii* and 38 *D. elator* contemporary samples.** Private alleles are those alleles not shared with any other subpopulation.
