# Supplementary material for: Comparison of genetic variation between rare and common congeners of Dipodomys with estimates of contemporary and historical effective population size
Source: PLoS One. 2022 Sep 13;17(9):e0274554. doi: 10.1371/journal.pone.0274554 (PMC9469943; doi:10.1371/journal.pone.0274554)
Supplement: S4 Table — (DOCX) [file pone.0274554.s010.docx]

| K | Reps | Mean LnP(K) | Stdev LnP(K) | Ln'(K) | \|Ln''(K)\| | Delta K |
| --- | --- | --- | --- | --- | --- | --- |
| 1 | 3 | -41942.7 | 4.6608 | NA | NA | NA |
| 2 | 3 | -41247.2 | 491.178 | 695.5667 | 22.43333 | 0.045673 |
| 3 | 3 | -40529.2 | 125.8167 | 718 | 944.7667 | 7.509069 |
| 4 | 3 | -40755.9 | 548.7333 | -226.767 | 419.5 | 0.764488 |
| 5 | 3 | -41402.2 | 816.379 | -646.267 | NA | NA |

**Table S4. Log-likelihood and delta K values used in the Evanno method for *D. elator* STRUCTURE analysis.**
