# Supplementary material for: Comparison of genetic variation between rare and common congeners of Dipodomys with estimates of contemporary and historical effective population size
Source: PLoS One. 2022 Sep 13;17(9):e0274554. doi: 10.1371/journal.pone.0274554 (PMC9469943; doi:10.1371/journal.pone.0274554)
Supplement: S5 Table — Private alleles are those alleles not shared with any other subpopulation. Observed and expected heterozygosity are the proportion of loci that are heterozygous based on Hardy-Weinberg frequencies. Historical observed and expected heterozygosity for D. elator are 0.042 and 0.038, respectively. π is a measure of nucleotide diversity. FIS indicates the inbreeding coefficient. The heterozygosity found in the Wichita samples is very large compared to what was found in the species as a whole. This is likely an artefact of which SNPs were used in this comparison. The SNPs evaluated are shared among both D. elator and D. ordii, and D. ordii is a common species, so the SNPs evaluated would be expected to have greater levels of diversity than SNPs evaluated in just D. elator. (DOCX) [file pone.0274554.s011.docx]

| Missingness value (-r) | Analyzed SNPs | Group | Private alleles | Observed heterozygosity | Expected heterozygosity | π | F_IS_ |
| --- | --- | --- | --- | --- | --- | --- | --- |
| 0.95 | 3,724 | *D. ordii*  (Dickens) | 1 | 0.370 | 0.346 | 4.15 x10^-3^ | +0.111 |
|  |  | *D.elator*  *(*Wichita) | 0 | 0.368 | 0.350 | 4.21 x10^-3^ | +0.101 |
| 0.75 | 3,732 | *D. ordii*  (Dickens) | 2 | 0.333 | 0.356 | 4.27 x10^-3^ | +0.2 |
|  |  | *D.elator*  *(*Wichita) | 0 | 0.369 | 0.351 | 4.21 x10^-3^ | +0.099 |
| 0.5 | 7,948 | *D. ordii*  (Dickens) | 8 | 0.284 | 0.290 | 3.81 x10^-3^ | +0.154 |
|  |  | *D.elator*  *(*Wichita) | 1 | 0.440 | 0.394 | 5.05 x10^-3^ | +0.107 |
| 0.25 | 13,089 | *D. ordii*  (Dickens) | 36 | 0.264 | 0.136 | 2.66 x10^-3^ | +0.004 |
|  |  | *D.elator*  *(*Wichita) | 65 | 0.536 | 0.373 | 5.79 x10^-3^ | +0.071 |

**Table S5. General summary statistics calculated in Stacks for a comparison between 3 individuals from each species that were collected in proximity (i.e. same tract of land).**
